# Double burden of malnutrition among school-aged children and adolescents: evidence from a community-based cross-sectional survey in two Nigerian States

**DOI:** 10.12688/aasopenres.13257.1

**Published:** 2021-07-21

**Authors:** Adeleye Adeomi, Adesegun Fatusi, Kerstin Klipstein-Grobusch

**Affiliations:** 1 Community Health, College of Health Sciences, Obafemi Awolowo University, Ile-Ife, Osun State, 220, Nigeria; 2 Division of Epidemiology and Biostatistics, School of Public Health, Faculty of Health Sciences, University of the Witwatersrand, Johannesburg, South Africa; 3 School of Public Health, University of Medical Sciences, Ondo, Ondo State, Nigeria; 4 Julius Global Health, Julius Center for Health Sciences and Primary Care, University Medical Center Utrecht, Utrecht University, Utrecht, The Netherlands

**Keywords:** Under-nutrition, Over-nutrition, Double burden of Malnutrition, School-aged children, Adolescents, Stunting, Underweight, Thinness

## Abstract

**Background: **Double burden of malnutrition (DBM) is the co-existence of overweight/obesity and undernutrition. Rising prevalence rates of childhood overweight/obesity in Nigeria have been reported, whilst undernutrition continues to be prevalent. This study aimed to estimate the prevalence and distribution of underweight, stunting, thinness, overweight/obesity, and DBM among school-aged children and adolescents in two Nigerian States.

**Methods: **This was a community-based cross-sectional study carried out in Osun and Gombe States. A total of 1,200 children aged 6 – 19 years were recruited using multi-stage sampling technique. Weight, height and data on demographic, socio-economic, household/family characteristics of the children were collected using structured interviewer administered questionnaires. Nutritional status was calculated using the WHO 2007 reference values using BMI-for-age (thinness, overweight/obesity), height-for-age (stunting) and weight-for-age (underweight). DBM was described at the population and individual levels.

**Results: **The mean age of the respondents was 11.6 ± 3.8 years. The overall prevalence rate of stunting was 34.9%, underweight was 13.5%, thinness was 10.3% and overweight/obese was 11.4% and 4.0% had individual level DBM, which typifies the DBM at individual and population levels. These rates differed significantly across demographic, socio-economic and household/family characteristics (p < 0.05). Gombe State, which is in the Northern part of Nigeria, had significantly higher burden of stunted, underweight and thin children than Osun State, while Osun State, in the Southern part of Nigeria, had a significantly higher burden of overweight/obesity.

**Conclusions: **The study found evidence of DBM both at population and individual levels. The overall prevalence rates of stunting, underweight, thinness and overweight/obesity in this study were high, and they differed significantly across the demographic, socio-economic and household/family characteristics. There is the need for government and all other stakeholders to design nutritional educational programmes that will target both under- and over-nutrition among older children in the different contexts.

## Introduction

Decades ago, the world faced the devastating burden of undernutrition among children, such that the term malnutrition was used almost exclusively to mean undernutrition ^1^. In recent years, there has been a steady rise in the prevalence of overweight and obesity among children and adolescents in all parts of the world, and the rate is increasing faster in low- and middle-income countries (LMIC) compared to high-income countries ^2^. Yet, undernutrition remains a major challenge in many LMICs; as such, many LMICs face a double burden of malnutrition (DBM) problems in terms of the co-existence of undernutrition and over-nutrition (overweight/obesity) ^3, 4^. The increasing incidence of overweight/obesity in LMICs has been attributed mainly to nutrition transition with changes in dietary patterns from a traditional to a westernized diet – “a diet high in saturated fat, sugar, and refined foods and low in fibre” – 5 and a reduction in energy expenditure fuelled by increasing sedentary activity patterns ^6^. DBM significantly and negatively impact population health and well-being as well as economic development in LMICs ^7^.

Nigeria, like many other LMICs, is currently undergoing nutritional transition ^8, 9^, with some researchers already reporting rising prevalence rates of overweight/obesity close to the global average of 18% among adolescents ^2, 10, 11^. On the other hand, the United Nations Children Fund (UNICEF) estimates that 33% of Nigerian children are stunted, and this has been corroborated by other studies ^12– 15^. Few studies in Nigeria are already reporting the existence of the double burden of malnutrition ^16– 18^, but these studies are scattered, limited in geographical distriution and mainly school based. There is, to date, no nationally representative community-based data on the nutritional status of children 6 – 14 years in Nigeria. The Nigeria Demographic and Health Survey (NDHS), the Multiple Indicator Cluster Survey (MICS) and the Nutrition and Health Survey, which all have nationally representative data on the nutritional status of children in Nigeria include only under-five children, and those 15 years and older ^19– 21^.

Also, although several studies have been carried out in Nigeria on the nutritional status of school-aged children and adolescents, many of the studies have the challenge of small sample size and comparability between survey findings has been challenging as different anthropometric indicators, reporting systems, cut-off points and reference values have been used ^22^. Another challenge is the geographical imbalance in the study locations of existing studies, with majority of the studies reported in settings with higher socio-economic status (ie the Southern and urban parts of Nigeria) ^22^. Furthermore, most of the existing studies in Nigeria have been school-based studies and out-of-school children, who are from economically poorer and more disadvantaged backgrounds, are thereby excluded. This study aims to address some of the existing shortcomings and research gaps relating to DBM among children and adolescents in Nigeria. Specifically, this study aimed to estimate the prevalence and distribution of under- and over-nutrition among school-aged children and adolescents in two Nigerian States from the northern and southern parts of the country, using a community-based approach.

## Methods

### Study design, population and size

This community-based cross-sectional study was carried out in Osun and Gombe States, located in the southern and northern parts of Nigeria respectively. The selection of the states was based on the wealth index as published by the NDHS ^19^. The wealth index was categorized according to geo-political zones. Therefore, one state each from the zones with lowest (North-East; Gombe State) and highest wealth (South-West; Osun State) index were selected randomly. The study population were school-aged children and adolescents aged between 6 and 19 years living in selected communities in the study locations. The mothers of the selected school-aged children and adolescents were also included in the study. Acutely ill children, those with chronic diseases that can affect their weight (like sickle cell diseases and cancers), those living with disabilities that affect their abilities to stand properly, and children whose mothers were not available even after a revisit on another day during the time of the survey were excluded from the study. The sample size was calculated using STATCALC on Epi-Info software ^23^. The prevalence of stunting among Nigerian children according to UNICEF (33%) ^4^ was used as the expected outcome, the acceptable margin of error was set at 5%, and the design effect was taken as 1.5. The design effect is the increase of sample size because of the hierarchical sampling model used in this study. After correcting for an anticipated non-response rate of 10%, the sample size came to 561 and was rounded off to 600 for each state, making a total of 1,200.

### Sampling technique

The 1,200 respondents were selected using a multi-stage sampling. Four Local Government Areas (LGAs), 8 wards and 40 enumeration areas (EAs) were selected in the first, second and third stages using simple random sampling technique (balloting method). At the fourth stage, 30 households were selected from each of the EAs using systematic random sampling technique, making a total of 1,200 households. At the household level (fifth stage), only one child/adolescent was randomly selected (using balloting) from all the eligible children and/or adolescents in the household. This is because children within the same household would probably have very similar exposures, especially at this level, potentially confounding the associations.

### Data collection

Ten research assistants and one field supervisor were recruited and trained in each of the 2 states. They were fluent in both the native and English languages and familiar with the sociocultural and geographical terrains of the study locations. The assistants and supervisors were trained for five days by the one of the authors (AAA). After the training, measurements were taken by the researcher and each of the research assistants on the same set of subjects to ensure minimal or no inter-tester and intra-tester errors. This exercise was repeated at intervals during the entire study to further assure quality control.

The questionnaires were interviewer-administered by use of REDCap ^24^. The questionnaire collected information on demographic, socio-economic and household/family characteristics, using relevant questions that were adapted from the NDHS ^19^. Anthropometric measurements of children/adolescents were taken according to standard protocols recommended by the International Society for the Advancement of Kinanthropometry ^25^. Weight was measured in 0.1 kilograms by the use of an Omron® electronic bathroom weighing scale. Height was measured to the nearest 0.1 meter using a stadiometer. Weighing scales were routinely standardized by use of known weights.

### Measurement of the outcome and explanatory variables

The outcome variable **,**nutritional status of the school-aged children and adolescents, was assessed using WHO 2007 reference values ^26^. Those with BMI-for-age Z-scores <-2, -2 to 1, > 1 to 2 and > 2 were classified as thinness, normal, overweight and obesity respectively. Weight-for-age and height-for-age Z scores < -2 were also used to assess underweight and stunting. Population level DBM signified communities with both under- and over-nutrition, while individual DBM was defined as children who were both stunted and overweight/obese.

The explanatory variables were States (Osun/Gombe), residence (rural/urban), age groups in years (6 – 9/10 – 13/14 – 16/17 – 19 years), sex (male/female), ethnic groups (Yoruba/Igbo/Hausa/Fulani/Minorities), household wealth index (poorest/poorer/middle/richer/richest), family type (monogamous/polygamous), mother’s education (less than secondary/secondary or more) and mother’s occupation (unemployed/employed). The wealth index was calculated using ownership of some household possessions, as was used by the NDHS ^19^. Principal component analysis was used to produce a common factor score for each household. The scores were divided into quintiles to categorize the households into poorest, poorer, middle, richer and richest households. The wealth index was later re-grouped into three categories (poorest/middle/richest) for the multi-variate analysis. 

### Data analysis

The data were analyzed using STATA version 15.1. The level of significance was set with a two-sided p-value less than 0.05. The distribution of thinness, underweight, stunting, overweight/obesity and individual DBM according to the demographic, socio-economic and household/family characteristics was done. Pearson’s chi-square test was used to test for associations at the bivariate level. The multinomial logistic regression was used to identify predictors of under-and over-nutrition at the population level, using the BMI-for-age categories as the dependent variable (i.e. (1) underweight (2) normal (3) overweight/obesity) with the comparator being the “normal” category.

### Ethical considerations

Ethical clearance was obtained from the Human Research Ethics Committee of the University of the Witwatersrand (certificate No: M190514) and relevant institutional review boards (IRB) in Osun (certificate No: OSHREC/PRS/569T/155) and Gombe (certificate No: MOH/ADM/621/1/142) States. Community entry was undertaken with advocacy visits to leaders and gate-keepers in selected communities. Participation was voluntary and the information volunteered was confidential as all questionnaires were coded. Participants were free to opt-out at any time in the study. Written consent was obtained from the parents and adolescents who were 18 years and above, while assent was obtained from those less than 18 years. All severely malnourished children, with BMI-for-age, weight-for-age or height-for-age Z-score less than -3, were referred to the nearby public health facilities for nutritional counselling and further management.

## Results

A total of 1200 school-aged children and adolescents – 601 (50.1%) males and 599 (49.9%) females – participated in the study, giving a male-to-female ratio of approximately 1:1. The mean age of the respondents was 11.6 ± 3.8 years.

Table 1 shows the prevalence and distribution of the nutritional status of the respondents according to their age and sex. Among the males, the prevalence of thinness was highest among those 15 years old (16.4%), followed by those 6 or 9 years in age (11.9%). The prevalence of overweight and obesity was highest among the younger male children with the highest prevalence being 23.5% (9 years) and 31.6% (8 years) for overweight and obesity respectively. Beyond the age of 13 years, nearly no male child was overweight nor obese. For the females, the prevalence of thinness was highest among the 6- and 10-year-old children (12.5%), while prevalence of overweight and obesity were highest among 11- and 7-year old children (20.6% and 33.3%) respectively. Unlike the males however, there were overweight females after the age of 13 years. On the whole, the prevalence of malnutrition was significantly different between males and females; 11.1% of males compared to 9.3% of females had thinness, while 5.7% of males and 10.5% of females were overweight and 3.2% of males and 3.5% of males were obese. The overall prevalence of malnutrition was 10.2% for thinness, 8.1% for overweight and 3.3% for obesity. 

**Table 1.** Age and sex distribution of the prevalence of thinness, overweight and obesity ^+^.

Age	Males (%)				Females (%)				Total (%)				P -value
	^a^n	Thinness	Over- weight	Obese	^a^n	Thinness	Over- weight	Obese	^a^N	Thinness	Over- weight	Obese	
6	46 (7.7)	8 (11.9)	4 (11.8)	1 (5.3)	58 (9.7)	7 (12.5)	2 (3.2)	2 (9.5)	104 (8.7)	15 (12.2)	6 (6.2)	3 (7.5)	^b^0.540
7	41 (6.8)	4 (6.0)	4 (11.8)	3 (15.8)	47 (7.8)	8 (14.3)	1 (1.6)	7 (33.3)	88 (7.3)	12 (9.8)	5 (5.2)	10 (25.0)	^b^0.209
8	58 (9.7)	6 (9.0)	6 (17.6)	6 (31.6)	43 (7.2)	1 (1.8)	10 (15.9)	1 (4.8)	101 (8.4)	7 (5.7)	16 (16.5)	7 (17.5)	^b^0.049 ^*^
9	53 (8.8)	4 (6.0)	8 (23.5)	1 (5.3)	61 (10.2)	6 (10.7)	11 (17.5)	2 (9.5)	114 (9.5)	10 (8.1)	19 (19.6)	3 (7.5)	^b^0.872
10	72 (12.0)	8 (11.9)	4 (11.8)	1 (5.3)	56 (9.3)	7 (12.5)	5 (7.9)	3 (14.3)	128 (10.7)	15 (12.2)	9 (9.3)	4 (10.0)	^b^0.479
11	33 (5.5)	2 (3.0)	0 (0.0)	4 (21.1)	44 (7.3)	2 (3.6)	13 (20.6)	1 (4.8)	77 (6.4)	4 (3.3)	13 (13.4)	5 (12.5)	^b^< 0.001 ^*^
12	59 (9.8)	3 (4.5)	4 (11.8)	2 (10.5)	52 (8.7)	5 (8.9)	1 (1.6)	3 (14.3)	111 (9.3)	8 (6.5)	5 (5.2)	5 (12.5)	^b^0.437
13	44 (7.3)	3 (4.5)	2 (5.9)	1 (5.3)	44 (7.3)	2 (3.6)	1 (1.6)	2 (9.5)	88 (7.3)	5 (4.1)	3 (3.1)	3 (7.5)	^b^0.827
14	41 (6.8)	4 (6.0)	0 (0.0)	0 (0.0)	38 (6.3)	5 (8.9)	5 (7.9)	0 (0.0)	79 (6.6)	9 (7.3)	5 (5.2)	0 (0.0)	^b^0.017
15	43 (7.2)	11 (16.4)	1 (2.9)	0 (0.0)	42 (7.0)	4 (7.1)	4 (6.3)	0 (0.0)	85 (7.1)	15 (12.2)	5 (5.2)	0 (0.0)	^b^0.066
16	23 (3.8)	2 (3.0)	0 (0.0)	0 (0.0)	24 (4.0)	4 (7.1)	2 (3.2)	0 (0.0)	47 (3.9)	6 (4.9)	2 (2.1)	0 (0.0)	^b^0.160
17	39 (6.5)	7 (10.4)	0 (0.0)	0 (0.0)	42 (7.0)	2 (3.6)	2 (3.2)	0 (0.0)	81 (6.8)	9 (7.3)	2 (2.1)	0 (0.0)	^b^0.047 ^*^
18	30 (5.0)	4 (6.0)	1 (2.9)	0 (0.0)	32 (5.3)	2 (3.6)	5 (7.9)	0 (0.0)	62 (5.2)	6 (4.9)	6 (6.2)	0 (0.0)	^b^0.172
19	19 (3.2)	1 (1.5)	0 (0.0)	0 (0.0)	16 (2.7)	1 (1.8)	1 (1.6)	0 (0.0)	35 (2.9)	2 (1.6)	1 (1.0)	0 (0.0)	^b^0.443
**Total**	601 (50.1)	67 (11.1)	34 (5.7)	19 (3.2)	599 (49.9)	56 (9.3)	63 (10.5)	21 (3.5)	1200 (100.0)	123 (10.2)	97 (8.1)	40 (3.3)	0.016 ^*^

* Statistically significant ^+^ The WHO BMI-for-age classification was used such that those with < -2, -2 to 1, > 1 to 2 and > 2 were categorized as thinness, normal, overweight and obesity respectively ^a^ - number in the group ^b ^test of association between male and female rates (Pearson chi-square test)

The distribution of the indicators of under- and over-nutrition according to States, residence, age groups, sex, ethnicity groups, household wealth index, family types, mother’s education and occupation is shown in Table 2. The overall prevalence rate of stunting was 34.9%, underweight was 13.5%, thinness was 10.3%, overweight/obese was 11.4% and 4.0% had individual level DBM in the study. Stunting was significantly associated with all the explanatory variables considered (p < 0.01), except residence (p = 0.672). Underweight was significantly associated with all explanatory variables, except residence (p = 0.227) and sex (p = 0.554), while BMI-for-age categories (thinness, normal and overweight/ obesity) were significantly associated with all the considered explanatory variables (p < 0.05), except family type (p = 0.667). Individual DBM was significantly associated with the State, residence, ethnicity, family type and mother’s occupation (p < 0.05). 

**Table 2.** Indicators of under- and over-nutrition and the associated factors.

	Height-for-Age		^‡^Weight-for-Age		BMI-for-Age			Individual DBM	
	Stunted	Otherwise	Under- weight	Otherwise	Thinness	Normal	Overweight/ Obesity	Present	Otherwise
**Overall Total **	419 (34.9)	781 (65.1)	72 (13.5)	463 (86.5)	123 (10.3)	940 (78.3)	137 (11.4)	48 (4.0)	1152 (96.0)
**State** Osun Gombe	136 (22.7) 283 (47.2)	464 (77.3) 317 (52.8)	22 (7.4) 50 (21.0)	275 (92.6) 188 (79.0)	40 (6.7) 83 (13.8)	464 (77.3) 476 (79.3)	96 (16.0) 41 (6.8)	36 (6.0) 12 (2.0)	564 (94.0) 588 (98.0)
	p < 0.001 *		p < 0.001 *		p < 0.001 *			**p < 0.001**	
**Residence** Rural Urban	206 (34.3) 213 (35.5)	394 (65.7) 387 (64.5)	38 (15.4) 34 (11.8)	209 (84.6) 254 (88.2)	77 (12.8) 46 (7.7)	481 (80.2) 459 (76.5)	42 (7.0) 95 (15.8)	16 (2.7) 32 (5.3)	584 (97.3) 568 (94.7)
	p = 0.672		p = 0.227		p < 0.001 *			p = 0.018 *	
**Age groups** 6 – 9 years 10 – 13 years 14 – 16 years 17 – 19 years	45 (11.1) 177 (43.8) 109 (51.7) 88 (49.4)	362 (88.9) 227 (56.2) 102 (48.3) 90 (50.6)	41 (10.1) 31 (24.2)	366 (89.9) 97 (75.8)	44 (10.8) 32 (7.9) 30 (14.2) 17 (9.6)	294 (72.2) 325 (80.4) 169 (80.1) 152 (85.4)	69 (17.0) 47 (11.6) 12 (5.7) 9 (5.1)	12 (2.9) 20 (5.0) 11 (5.2) 5 (2.8)	395 (97.1) 384 (95.0) 200 (94.8) 173 (97.2)
	p < 0.001 *		p < 0.001 *		p < 0.001 *			p = 0.309	
**Sex** Male Female	251 (41.8) 168 (28.0)	350 (58.2) 431 (72.0)	34 (12.6) 38 (14.3)	236 (87.4) 227 (85.7)	67 (11.1) 56 (9.3)	481 (80.0) 459 (76.6)	53 (8.8) 84 (14.0)	18 (3.0) 30 (5.0)	583 (97.0) 569 (95.0)
	p < 0.001 *		p = 0.554		p = 0.014 *			p = 0.075	
**Ethnicity** Yoruba Igbo Hausa Fulani Minorities	165 (25.8) 12 (31.6) 73 (48.7) 72 (49.0) 97 (42.9)	474 (74.2) 26 (68.4) 77 (51.2) 75 (51.0) 129 (57.1)	22 (7.2) 0 (0.0) 12 (22.6) 22 (33.3) 16 (17.2)	283 (92.8) 18 (100.0) 41 (77.4) 44 (66.7) 77 (82.8)	41 (6.4) 2 (5.3) 15 (10.0) 27 (18.4) 38 (16.8)	506 (79.2) 34 (89.5) 127 (84.7) 106 (72.1) 167 (73.9)	92 (14.4) 2 (5.3) 8 (5.3) 14 (9.5) 21 (9.3)	37 (5.8) 1 (2.6) 3 (2.0) 3 (2.0) 4 (1.8)	602 (94.2) 37 (97.4) 147 (98.0) 144 (98.0) 222 (98.2)
	p < 0.001 *		p < 0.001 *		p < 0.001 *			p = 0.022 *	
**Household** **wealth index** Poorest Poorer Middle Richer Richest	113 (47.1) 83 (34.4) 80 (32.1) 73 (30.9) 70 (29.9)	127 (52.9) 158 (65.6) 169 (67.9) 163 (69.1) 164 (70.1)	26 (25.5) 19 (16.2) 15 (13.4) 11 (10.0) 1 (1.1)	76 (74.5) 98 (83.8) 97 (86.6) 99 (90.0) 93 (98.9)	45 (18.8) 28 (11.6) 18 (7.2) 24 (10.2) 8 (3.4)	169 (70.4) 180 (74.7) 208 (83.5) 191 (80.9) 192 (82.1)	26 (10.8) 33 (13.7) 23 (9.2) 21 (8.9) 34 (14.5)	13 (5.4) 10 (4.1) 6 (2.4) 12 (5.1) 7 (3.0)	227 (94.6) 231 (95.9) 243 (97.6) 224 (94.9) 227 (97.0)
	p < 0.001 *		p < 0.001 *		p < 0.001 *			p = 0.373	
**Family type** Monogamous Polygamous	346 (32.5) 73 (53.7)	718 (67.5) 63 (46.3)	54 (11.2) 18 (33.3)	427 (88.8) 36 (66.7)	107 (10.1) 16 (11.8)	833 (78.3) 107 (78.7)	124 (11.7) 13 (9.6)	45 (4.2) 3 (2.2)	1019 (95.8) 133 (97.8)
	p < 0.001 *		p < 0.001 *		p = 0.667			p < 0.001 *	
**Mother’s** **education** < Secondary ≥ Secondary	155 (41.4) 264 (32.0)	219 (58.6) 562 (68.0)	40 (24.5) 32 (8.6)	123 (75.5) 340 (91.4)	61 (16.3) 62 (7.5)	283 (75.7) 657 (79.5)	30 (8.0) 107 (13.0)	12 (3.2) 36 (4.4)	362 (96.8) 790 (95.6)
	p = 0.001 *		p < 0.001 *		p < 0.001 *			p = 0.346	
**Mother’s** **Occupation** Unemployed Employed	105 (42.9) 314 (32.9)	140 (57.1) 641 (67.1)	26 (23.4) 46 (10.8)	85 (76.6) 378 (89.2)	47 (19.2) 76 (8.0)	178 (72.7) 762 (79.8)	20 (8.2) 117 (12.3)	3 (1.2) 45 (4.7)	242 (98.8) 910 (95.3)
	p = 0.003 *		p = 0.001 *		p < 0.001 *			p = 0.013 *	

*statistically significant

Table 3 shows the factors associated with under-nutrition (thinness) and over-nutrition (overweight/obesity) after controlling for all the explanatory variables using multinomial regression analysis. The odds for thinness were significantly higher among minority ethnic groups (OR: 2.63, p = 0.040, 95% CI: 1.04 to 6.62) and children with unemployed mothers (OR: 1.64, p = 0.047, 95% CI: 1.01 to 2.68), and lower among those from middle (OR: 0.55, p = 0.016, 95% CI: 0.34 to 0.89) and richest households (OR: 0.25, p = 0.002, 95% CI: 0.10 to 0.60). Urban dwellers (OR: 2,63, p < 0.001, 95% CI: 1.68 to 4.61) and females (OR: 1.63, p = 0.011, 95% CI: 1.12 to 2.45) had increased odds for being overweight/obese, while those in the middle wealth index category (OR: 0.56, p = 0.045, 95% CI: 0.32 to 0.99), age groups 10 – 13 years (OR: 0.61, p = 0.020, 95% CI: 0.40 to 0.90), 14 – 16 years (OR: 0.32, p = 0.001, 95% CI: 0.17 to 0.63) and 17 – 19 years (OR: 0.31, p = 0.002, 95% CI: 0.15 to 0.64) had lower odds of being overweight/obese. 

**Table 3.** Factors associated with malnutrition among school-aged children and adolescents in two Nigerian States using multinomial logistic regression analysis.

^a^THINNESS								
Variables	Empty Model				Full Model			
	OR	p-value	95% CI		aOR	p-value	95% CI	
			Lower	Upper			Lower	Upper
**State** Gombe (R) Osun	0.49	0.001 *	0.33	0.74	1.05	0.910	0.42	2.62
**Residence** Rural (R) Urban	0.63	0.018 *	0.43	0.92	0.79	0.280	0.51	1.22
**Age groups** 6 – 9 years (R) 10 – 13 years 14 – 16 years 17 – 19 years	0.66 1.19 0.75	0.089 0.504 0.336	0.41 0.72 0.41	1.07 1.96 1.35	0.66 1.15 0.65	0.104 0.612 0.178	0.40 0.67 0.35	1.09 1.96 1.22
**Sex** Male (R) Female	0.88	0.491	0.60	1.28	0.91	0.619	0.61	1.34
**Ethnicity** Yoruba (R) Igbo Hausa Fulani Minorities	0.76 1.47 3.25 2.82	0.714 0.226 <0.001 * <0.001 *	0.18 0.79 1.92 1.75	3.29 2.75 5.50 4.55	1.01 0.99 1.61 2.63	0.996 0.988 0.381 0.040 *	0.21 0.34 .56 1.04	4.64 2.92 4.65 6.62
**Wealth index** Poorest (R) Middle Richest	0.45 0.16	<0.001 * <0.001 *	0.30 0.07	0.69 0.34	0.55 0.25	0.016 * 0.002 *	0.34 0.10	0.89 0.60
**Family type** Monogamous (R) Polygamous	1.16	0.597	0.66	2.04	0.82	0.508	0.45	1.49
**Mother’s education** < Secondary (R) ≥ Secondary	0.44	<0.001 *	0.30	0.64	0.77	0.294	0.47	1.26
**Mother’s Occupation** Employed (R) Unemployed	2.51	<0.001 *	1.68	3.74	1.64	0.047 *	1.01	2.68
^a^OVERWEIGHT								
**State** Gombe (R) Osun	2.40	<0.001 *	1.63	3.54	2.30	0.057	0.98	5.11
**Residence** Rural (R) Urban	2.37	<0.001 *	1.61	3.48	2.63	<0.001 *	1.68	4.61
**Age groups** 6 – 9 years (R) 10 – 13 years 14 – 16 years 17 – 19 years	0.62 0.30 0.25	0.018 * <0.001 * <0.001 *	0.41 0.16 0.12	0.92 0.57 0.52	0.61 0.32 0.31	0.020 * 0.001 * 0.002 *	0.40 0.17 0.15	0.90 0.63 0.64
**Sex** Male (R) Female	1.66	0.007 *	1.15	2.40	1.63	0.011 *	1.12	2.45
**Ethnicity** Yoruba (R) Igbo Hausa Fulani Minorities	0.47 0.28 0.53 0.58	0.219 0.002 * 0.056 0.042 *	0.14 0.13 0.27 0.35	1.56 0.62 1.02 0.98	0.58 0.66 0.80 1.35	0.405 0.496 0.707 0.536	0.16 0.20 0.25 0.52	2.09 2.17 2.56 3.48
**Wealth index** Poorest (R) Middle Richest	0.86 1.15	0.549 0.617	0.54 0.66	1.39 2.00	0.56 0.61	0.045 * 0.151	0.32 0.31	0.99 1.20
**Family type** Monogamous (R) Polygamous	0.82	0.511	0.45	1.50	1.35	0.386	0.68	2.68
**Mother’s education** < Secondary (R) ≥ Secondary	11.54	0.049 *	1.00	2.36	1.05	0.869	0.61	1.81
**Mother’s Occupation** Employed (R) Unemployed	0.63	0.087	0.37	1.07	0.98	0.937	0.53	1.80

*Statistically significant OR – odds ratio; aOR – adjusted odds ratio; CI – confidence interval; R – reference value ^a ^Base outcome or comparator was the “normal” category

## Discussion

The findings of the present study show that the overall prevalence rates for under- and over-nutrition were high, typifying the DBM at the population level. The rate for stunting was 34.9%, underweight was 13.5% and thinness was 10.3%, while at the same time 11.4% were overweight/obesity in the same population. The DBM was also found at the individual level, with 4% of the children being stunted and overweight/obese at the same time. Although, much attention has not been given to DBM in Nigeria previously, recent studies have similarly reported the DBM at population and individual levels among different Nigerian populations ^16– 18^. This is important because most nutritional interventions for Nigerian children before now have focused on undernutrition alone, but with the current knowledge, attention must be paid to both under- and over-nutrition. 

Using the BMI-for-age categories, undernutrition (thinness) and over-nutrition (overweight/obesity) rates were 10.2% and 11.4% respectively. Most of the previous studies carried out in Nigeria that used BMI-for-age categories, especially those carried out more than 5 years ago, reported thinness being considerably higher than overweight/obesity ^27– 30^. Omigbodun *et al.* ^28^ who conducted a similar study among adolescents in southwestern Nigeria in 2010, for example, reported 18.9% and 2.3% as prevalence rates for thinness and overweight/obesity respectively, while Fetuga *et al.* ^30^ reported 22.2% and 3.5% respectively for a similar study among school-aged children in 2011. The present study however, is reporting a slightly higher prevalence of overweight/obesity than that of thinness, with about a fifth of children in Osun State, urban dwellers and school-aged children being overweight/obese. These results are in line with recent Nigerian studies by Asiegbu *et al.* in 2017 ^31^, Onoja *et al.* in 2019 ^32^ and Olasinde *et al.* in 2020 ^33^ all reporting higher rates of overweight/obesity than thinness among children 6 – 19 years.

The prevalence of childhood overweight/obesity has been increasing globally, and Nigeria is not exempted. UNICEF reported increases from 2% to 8% for Nigeria within 2 years from 2017 ^4^ to 2019 ^34^. The overall prevalence of overweight/obesity in this study (11.4%) is higher than what UNICEF reported for Nigeria and sub-Sahara Africa ^34^. The prevalence rates in Osun State for children 6 – 19 years old and those from the wealthiest households are already nearing the global average of 18% among adolescents ^34^. Similarly, recent Nigerian studies by Otuneye *et al.* ^10^ Omisore *et al.* ^35^ and Bello *et al.* ^36^ carried out among children 6 – 19 years from different parts of the country reported prevalence rates between 10.4% to 15.4% for overweight/obesity. This increasing prevalence of overweight/obesity among older children in Nigeria may be due to nutrition transition, with increased adoption of westernized diets and reduced energy expenditure, which has been reported in Nigeria ^8, 9^. 

On the other hand, the present study shows persistent under-nutrition among school-aged children and adolescents in the study population, and possibly in Nigeria as a whole. More than a third of the children in this study were stunted, which is more than the 33% reported by UNICEF ^37^. Particularly alarming is the prevalence rate of stunting, reaching nearly 50% in Gombe State, among older adolescents, in Hausa and Fulani ethnic groups and in the poorest households. The prevalence of underweight, measured using WFA reference values, was also a concern, with a third of Fulani children and a quarter of younger adolescents and children from the poorest wealth index being underweight. This paradoxical co-existence of overweight/obesity and undernutrition in the present study typifies the DBM (i.e. under- and over-nutrition) ^38^.

After controlling for possible confounding variables using the multivariate analysis, older children from the minority tribes had 3 times higher likelihood of thinness compared to those from the Yoruba ethnic group. Regional and ethnic variation in malnutrition among Nigerian children has been reported by previous researchers ^17, 39^. Similarly, those whose mothers were unemployed had 2 times higher likelihood for thinness compared to those with employed mothers. School-aged children and adolescents in the middle wealth index group had 45% less likelihood while those in the riches wealth index group had 75% less likelihood of thinness than those in the poorest wealth index group. This finding further underscores the importance of socio-economic and household factors in the nutritional status of the child, as has been previously reported by other studies in Nigeria and sub-Saharan Africa ^19, 40, 41^. 

Concerning the predictors of overweight/obesity, urban dwellers were 3 times more likely to be overweight/obese than rural dwellers. Urban-rural differentials in childhood malnutrition has been previously established ^32, 35, 40^. Urbanization has been associated with nutrition transition, and hence with increasing prevalence of overweight/obesity ^8, 9^. Furthermore, females were 2 times more likely to be overweight/obese than boys, and the likelihood for overweight/obesity reduced with increasing age groups of the children and adolescents. The pattern of distribution of over-nutrition (overweight/obesity) across sex and age groups seems to be consistent in most of the studies, with overweight/obesity higher in the girls and younger age groups ^33, 42– 44^. The findings of the present study corroborated this pattern, with the children 6 – 9 years and girls having significantly higher prevalence rates of overweight/obesity. 

A limitation of this study is that it cannot not be generalized to all of Nigeria, because only 2 out of 36 states were involved in the study. The cross-sectional nature of the study also means that causality cannot be determined in this study. Lastly, this study did not set out to exhaust the many complex explanatory factors for both under-nutrition and over-nutrition among school-aged children and adolescents. 

## Conclusion

The study found evidence of DBM both at population and individual levels. The overall prevalence rate of stunting was 34.9%, underweight was 13.5%, thinness was 10.3%, overweight/obese was 11.4% and 4.0% had individual level DBM in the study. These rates differed significantly across the demographic, socio-economic and household/family characteristics. There is the need for government and all other stakeholders to design nutritional educational programmes that will target both under- and over-nutrition among older children in the different contexts.

## Data availability

Dryad. Double burden of Malnutrition in two Nigerian States. DOI: https://doi.org/10.5061/dryad.qnk98sfh0 (Adeomi, Adeleye (2021)).

Data are available under the terms of the Creative Commons Zero "No rights reserved" data waiver (CC0 1.0 Public domain dedication).
