# Supplementary material for: Double burden of malnutrition among school-aged children and adolescents: evidence from a community-based cross-sectional survey in two Nigerian States
Source: AAS Open Res. 2021 Jul 21;4:38. [Version 1] doi: 10.12688/aasopenres.13257.1 (PMC8546731; doi:10.12688/aasopenres.13257.1)
Supplement: Peer review history

# Peer review: Reviewer response for version 1

**DOI:** 10.21956/aasopenres.14379.r28932

**Published:** 2021-10-25

**Authors:** Nelia Steyn

**Affiliations:** 1 Department of Human Biology, University of Cape Town, Cape Town, South Africa

A well-written comprehensive article. Clear aims and well described findings. I only have these comments:

1. When mentioning cut-off points, add SD e.g. -2SD, -1SD.
2. The age categories are not clearly defined in the methods section.
3. Describe the adjustments made in Table 3.
4. The discussion should include more data on other African neighbouring countries which have a DBD.

Is the work clearly and accurately presented and does it cite the current literature?

Yes

If applicable, is the statistical analysis and its interpretation appropriate?

Yes

Are all the source data underlying the results available to ensure full reproducibility?

Yes

Is the study design appropriate and is the work technically sound?

Yes

Are the conclusions drawn adequately supported by the results?

Yes

Are sufficient details of methods and analysis provided to allow replication by others?

Yes

Reviewer Expertise:

Nutrition and double burden of disease; Nutritional status of children

I confirm that I have read this submission and believe that I have an appropriate level of expertise to confirm that it is of an acceptable scientific standard.

# Peer review: Reviewer response for version 1

**DOI:** 10.21956/aasopenres.14379.r28805

**Published:** 2021-08-24

**Authors:** Florence Basiimwa Tushemerirwe

**Affiliations:** 1 School of Public Health, New Mulago Hospital Complex, Makerere University College of Health Sciences, Kampala, Uganda

**General feedback – **The publication is well written, I enjoyed reading it. Below are my minor suggestions for improvement.

****

**Abstract background:**

- The rising prevalence of overweight/obesity rates in Nigeria – from what proportion and when, to what proportions and when? Is it possible to quote the change?

**Abstract conclusion** – overall prevalence rates were high – compared to what? Is there a standard to compare with at that stage?

**Introduction:**

Paragraph one:

- Second sentence - I would propose to include some summary statistics, to show the increasing burden of Overweight/Obesity among children – also, to mention the period of the change.
- Last sentence – I propose not to start the sentence with an acronym.

**Methodology details:**

**Study sample size – **Please justify the use of the stunting indicator to estimate your sample size (e.g. was it the one that could generate a meaningful sample for the study, etc.). Please justify that choice.

**Data Collection:**

- Research Assistants training – what training did they receive?
- Please mention the period when the data was collected, so the readers can compare it with similar studies.

**Results** – Excellent data presentation.

**Discussion of results:**

 Paragraph one:

- Paragraph one - Opening sentence – "the…overall prevalence rates for under- and over-nutrition were high…" - compared to which standard? Please mention the acceptable rates (similar comments as in the introduction).
- Paragraph one – last sentence – please mention some of the interventions for Nigerian children here – or, give some references, for the reader to refer to.

Paragraph three:

- Last sentence, which reads: "This increasing prevalence of overweight/obesity among older children in Nigeria may be due to nutrition transition" etc. Is it only the nutrition transition? How about urbanization, globalization? Earlier, the authors mention the existence of unhealthy food (rich in calories, salt, sugar, and oils). Could there be a linkage to the global food trade?

**Study Limitations** – the authors state these very well. However, referring to the variables in the conceptual framework that guided the study, could it be possible to mention or relate the study findings to national studies on diet/food intake, food security, wealth inequalities that are determinants of childhood stunting in Nigeria? 

 Nwosu and Ataguba (2020 ^1^): explain the socio-economic inequalities and how these influence childhood stunting. 

**Areas for further research** – the authors could include areas for further research. The DBM is a serious public health problem, which other researchers in Nigeria confirmed. This study could open up areas for further investigation, as they already allude to this in the study limitations. I would propose explanatory qualitative studies would go a long way to generate local-context information to inform policy.

Is the work clearly and accurately presented and does it cite the current literature?

Yes

If applicable, is the statistical analysis and its interpretation appropriate?

Yes

Are all the source data underlying the results available to ensure full reproducibility?

Yes

Is the study design appropriate and is the work technically sound?

Yes

Are the conclusions drawn adequately supported by the results?

Yes

Are sufficient details of methods and analysis provided to allow replication by others?

Yes

Reviewer Expertise:

The Food Environment, Childhood overweight, and Obesity, Food systems,  Nutritional Epidemiology, Non-communicable diseases

I confirm that I have read this submission and believe that I have an appropriate level of expertise to confirm that it is of an acceptable scientific standard.

## References

[1] : Explaining changes in wealth inequalities in child health: The case of stunting and wasting in Nigeria. *PLoS One* .2020;15(9) : 10.1371/journal.pone.0238191 e0238191 10.1371/journal.pone.0238191 32925960PMC7489558
